# Computer-aided diagnosis of skin cancer based on soft computing techniques

**DOI:** 10.1515/med-2020-0131

**Published:** 2020-09-08

**Authors:** Zhiying Xu, Fatima Rashid Sheykhahmad, Noradin Ghadimi, Navid Razmjooy

**Affiliations:** Yuanpei College, Shaoxing University, Shaoxing, Zhejiang, 312000, China,; Young Researchers and Elite Club, Ardabil Branch, Islamic Azad University, Ardabil, Iran

**Keywords:** skin cancer, image segmentation, feature extraction, feature selection, convolutional neural networks, classification, satin bowerbird optimization

## Abstract

Skin cancer is a type of disease in which malignant cells are formed in skin tissues. However, skin cancer is a dangerous disease, and an early detection of this disease helps the therapists to cure this disease. In the present research, an automatic computer-aided method is presented for the early diagnosis of skin cancer. After image noise reduction based on median filter in the first stage, a new image segmentation based on the convolutional neural network optimized by satin bowerbird optimization (SBO) has been adopted and its efficiency has been indicated by the confusion matrix. Then, feature extraction is performed to extract the useful information from the segmented image. An optimized feature selection based on the SBO algorithm is also applied to prune excessive information. Finally, a support vector machine classifier is used to categorize the processed image into the following two groups: cancerous and healthy cases. Simulations have been performed of the American Cancer Society database, and the results have been compared with ten different methods from the literature to investigate the performance of the system in terms of accuracy, sensitivity, negative predictive value, specificity, and positive predictive value.

## 1 Introduction

Skin cancer is a high-prevalence disease in the United States, which occurs in the largest part of the body, i.e., the skin. Skin cancer usually develops on the outer layer of the skin, which may initially appear as a swelling, bump or other parts of the skin. Melanoma is considered one of the most malignant and deadly skin cancers worldwide. Although only about 1% of skin cancer is related to melanoma, it is the main reason of death among other skin cancer diseases [1]. In ref. [2], it has been reported that in 2020 there will be 1,00,350 new cases and 6,850 deaths due to melanoma in the United States. Researchers have found that skin cancer increases the likelihood of other cancers and hence early diagnosis of skin cancer is very important, which can significantly prevent death from this fatal cancer [3]. The two major problems in this regard are as follows:

(1) In most cases, skin lesions become malignant due to lack of attention to the skin lesions on their body surface or lack of access to experienced dermatologists.
(2) In many cases, skin lesions are misdiagnosed by physicians because of the high similarity of their characteristics.

Melanoma and Clark, for example, are two very similar skin lesions, except that melanoma is a malignant and deadly cancer and Clark is a benign skin lesion. Therefore, providing a method for the diagnosis of melanoma at an early stage is very useful and valuable [4]. In the last two decades, many studies have been performed on the rapid and accurate diagnosis of melanoma by dermoscopy images with diagnostic accuracy between 70% and 95% [5,6,7,8,9]. Recently, the use of machine vision and artificial intelligence as non-destructive tools in medical applications has been increasing. Besides, the significance of image processing in medical applications helps the physicians and radiologists to reduce the complexity and increase the early detection speed for disease diagnosis. One of the beneficial tools for medical cancer diagnosis is the artificial neural networks (ANNs). The ANN is a widely used methodology in artificial intelligence, which is inspired by the human brain interactions between synapses and neurons. The ANN method is a good black box-based tool for classification of the nonlinear problems with the least attempts [10,11,12,13,14,15,16]. In recent years, a new kind of neural network has been proposed based on deep learning, which is known as convolutional neural network (CNN). The CNN is often employed for image or speech analyses in machine learning. After the application of CNN in image processing, several researchers started to work on using CNN as a tool in medical image processing. For example, Sreelatha et al. [9] proposed a technique for melanoma diagnosis from dermoscopy images by the Gradient and Feature Adaptive Contour (GFAC) model. The study also used pre-processing and noise reduction to make the process faster and more accurate. The proposed GFAC model is a noise-free method. The method was then applied to the PH2 dataset. The method was then compared with different methods in the literature to indicate its effectiveness. Hekler et al. [17] proposed another skin cancer classification method based on artificial intelligence. The study applied dermoscopy images to classify it into five divisions. The method was based on the deep learning method to train a single CNN. The method was applied in a collection of 13 German university hospitals. The final results of the method were good.

Tschandl et al. [18] presented a classification method using CNN by training 7,895 dermoscopy images. The method was then performed to a set of 2,072 test cases, and the results were validated by some experts.

Tan et al. [19] proposed an optimized method for skin cancer detection based on an improved version of particle swarm optimization (PSO) algorithm. The PSO was used to optimize the feature extraction of the dermoscopy images. The method was then applied to some different benchmarks to show the PSO superiority in terms of performance toward some well-known methods.

**Figure 1:**
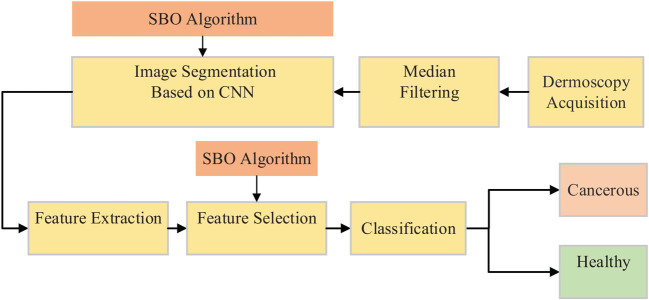
The flowchart of the proposed skin cancer diagnosis system.

The satin bowerbird optimization (SBO) algorithm is a new meta-heuristic algorithm that was first proposed by Musavi et al. [20]. The SBO algorithm was inspired by the method of making special nests by the male bowerbird to attract the females. In this study, an optimized comprehensive methodology is presented for skin cancer diagnosis in the dermoscopy images. The main contribution of the method is to use the CNN optimized by the SBO, which increases the accuracy of the network compared with the classic gradient descent method. The flowchart of the presented system is shown in Figure 1.

As shown in Figure 1, after receiving the input dermoscopy image, a preprocessing including median filtering has been applied to enhance the image quality by eliminating a part of the existing noises. In the next step, a new image segmentation based on the aforementioned optimized CNN has been applied to segment the skin-like area. In the next step, some features of the segmented area have been extracted for reducing the data complexity and easier classification. Here, an optimized feature selection has been used to select the useful features. Finally, after extracting the useful data from the image, a support vector machine (SVM) has been used for classifying the images into the following two groups: cancerous and healthy.

## 2 Materials and methods

All the required materials and methods that are used for skin cancer diagnosis in this research are as follows.

### 2.1 CNN

The CNN is a mostly used method based on deep learning in which multiple layers are trained in new ways. These networks are a kind of multilayer neural network designed for two-dimensional data such as image. Different parts of the image have been implemented as inputs to the neural network layers, which are hierarchical, and each layer is extracted by applying digital filters. Convolution layers convolve the image by using different kernels. The convolution process has the following three important features:

1. Sharing weights for the identical feature to decrease the parameter number.
2. Local learning connection.
3. Invariance according to the position of the object.

**Figure 2:**
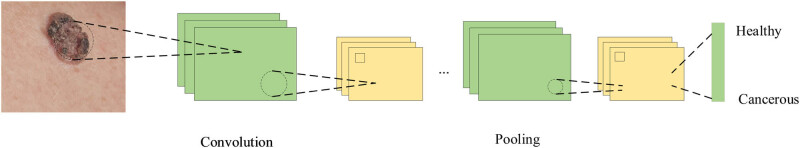
The structure of a CNN.

By considering the advantages provided by the convolution process, some research studies in the literature used it as an alternative to fully integrated layers to speed up the learning process. In general, the CNN contains three layers, including the convolutional layer, the pooling layer, and the fully connected layer, where different layers perform various tasks. The CNN architecture has several convolution layers that convolve the input image with filters whose output coefficients can be trained. These filters move over the image. Each separate filter creates a set of features, which consequently gives us the use of *n* filters of the set of features. Each filter has weights called *w*, which can be trained as filter coefficients. During the training, the network is sequenced, and the output obtained from the convolution operation is computed with a number that we consider to be a bias number and is stored in the feature plane. The pooling layer is usually followed by the convolution layer, which can be used to reduce the dimension of network features and parameters. Like the convolution layer, the pooling layer also uses adjacent pixels to compute. There are different strategies for pooling, but the most common way is to use the maximum pooling method. In the pooling method, blocks of the size 2 × 2 are considered and by moving these blocks over the image through 4 pixels, their maximum value is selected and transferred to the next layer that the number of features has been kept but its size has been reduced. Therefore, in the final step, we use fully connected layers, which actually convert the 2D feature into a one-dimensional vector. This layer accounts for 77% of the CNN parameters. Figure 2 shows the arrangement of the employed CNN.

The CNN has two levels of training: the feed-forward level and the backpropagation (BP) level. In the feed-forward level, the original image is injected to the network by applying a dot multiplication with the parameters of the neurons to provide convolutional operations on the layers.

Afterward, the output of the network is evaluated. For network training (set the network parameters), the output is employed to evaluate the network error rate. Then, the error rate of the network based on BP is calculated. In this stage, the chain rule is employed for evaluating the gradient of the parameters and the parameters change based on the error impact on the network. After updating the parameters, feed-forward is performed until the correct number of training. Here, local feature extraction is adopted for achieving the regional features of the input image. The learning is applied here to achieve some number of kernel matrices to extract the main characteristics of the dermoscopy skin cancer images. The present research adopts the BP technique for optimizing the weights of network connections. Sliding window is employed as a vector for implementing the convolution so that the weights and the dot product have been added up. For applying the activation function, rectified linear unit (ReLU) is used, i.e., *f*(*x*) = max(*x*, 0) [21]. For decreasing the output scale, Max pooling is used. In the present study, the subsequent layer of the sliding grid is considered as the highest value. After initializing the CNN, an optimization technique is required for error minimization of the actual output and the estimated output by varying the internal weights. Here, the BP algorithm is used for this purpose [6,22]. The main part of BP is to adopt the gradient descent algorithm for minimizing. The gradient descent is a minimization procedure on the cross-entropy loss [23]. The considered cost function for the explained cases is as follows:

(1) L=\mathop{\sum }\limits_{j=1}^{N}\mathop{\sum }\limits_{i=1}^{M}-{d}_{j}^{(i)}\hspace{.25em}\log \hspace{.25em}{z}_{j}^{(i)},

where {d}_{j}=\left(0,\ldots ,0,\mathop{\underbrace{1,\ldots 1}}\limits_{k},0,\ldots ,0\right) represents a vector with the desired output and {z}_{j} determines the softmax function of the *m*th class by the following equation:

(2) {z}_{j}^{(i)}=\frac{{e}^{{f}_{j}}}{\mathop{\sum }\limits_{i=1}^{M}{e}^{{f}_{i}}},

where *N* represents the sample quantity.

Therefore, by modifying the cost function based on the weight penalty with a coefficient *θ*,

(3) L=\mathop{\sum }\limits_{j=1}^{N}\mathop{\sum }\limits_{i=1}^{M}-{d}_{j}^{(i)}\hspace{.25em}\log \hspace{.25em}{z}_{j}^{(i)}+\frac{1}{2}\theta \sum _{K}\sum _{L}{W}_{K,L}^{2},

where *W*_*k*_ describes the weight of connection, *L* represents the total number of layer connections, and *K* is the number of layer *l* connections.

Although CNN has optimal arrangement for classification, most of the layouts are achieved experimentally that reduces its accuracy.

In recent years, some methods have been introduced to modify them based on bio-inspired techniques [24,25]. Metaheuristics are some kinds of optimization methods that have been inspired by different phenomena and have better efficiency in finding a global optimum value in less time. Recently, several types of metaheuristics have been introduced [26]. For instance, genetic algorithm [27] that simulates Darwin’s principles of selection to find the optimal formula, grass fibrous root optimization algorithm that simulates the fibrous root behavior [28], butterfly optimization algorithm [29] that simulates the butterflies’ migration from the cold areas to the warmer places in the cold seasons, and teaching–learning-based optimization algorithm [30] that simulates the relations between teaching and learning. In this study, another new metaheuristic method, called SBO algorithm, is employed for improving the CNN efficiency.

### 2.2 SBO algorithm

The bowerbirds are some kinds of interesting Australian birds which have a unique way for mating. In the mating season, the adult male bower builds his special bower by using his leathers, sparkling objects, flowers, fruits, and branches along with dramatic gestures to attract females. These parameters (the beauty of the bower and the male dramatic gestures) attract the females to the bower. These parameters form the main structure of the SBO algorithm [20]. The steps of the SBO algorithm are briefly described in the following.

#### 2.2.1 Initializing

Like any population-based metaheuristic, the SBO algorithm was initialized with a set of random population. The population in the SBO is the positions of the bowers such that each of them is an *n* dimensional vector of parameters of the problem that should be optimized. The bower parameters form the variables of the optimization problem. The bower’s attractiveness is a combination of these parameters. The initial population of the SBO can be defined as follows:

(4) {W}_{h}=({w}_{1},{w}_{2},\ldots ,{w}_{m}),

where *W*_*h*_ describes the *h*th solution and (*w*_1_, *w*_2_,…,*w*_*m*_) is the population of the solution.

The probability of fitness evaluation is mentioned as the attractiveness of the bower. Based on the bower probability theory, the male stain is selected by a female bowerbird. Similarly, after selecting a bower by the assigned probability of the male, it mimics the bower construction. Therefore

(5) {\text{Prob}}_{i}=\frac{{\text{fit}}_{i}}{\mathop{\sum }\limits_{n=1}^{NB}{\text{fit}}_{n}},

(6) {\text{fit}}_{i}=\left\{\begin{array}{l}\frac{1}{1+f({x}_{i})},{.25em}f({x}_{i})\ge 0\\ 1+|f({x}_{i})|,\hspace{.25em} f({x}_{i})\lt 0,\end{array}\right.

where \hspace{.25em}f({x}_{i}) describes the cost function value of the *i*th position.

#### 2.2.2 Elitism

The best solution of each stage is considered as elitism. Basically, each male satin bowerbird constructs his bower based on his natural instinct. Each satin bowerbird uses its instinct on its similarity of every other bird in the mating season to construct and beautification of his bower. In other words, although each male satin bowerbird uses material for decorating its bower, it benefits its experience as a key factor to attract lot of attention to his specific bower. In a word, we can say that experience has a significant effect on both construction of bower and dramatic gestures, which makes elder males to have more potential to attract more attention to their bower. In the algorithm, the position of the best bower is considered as iteration’s elite which has the capability of affecting other positions.

#### 2.2.3 Position updating

All of the variations at the bowers in every cycle of algorithm are performed based on the following equation:

(7) {W}_{hj}^{\text{new}}={W}_{hj}^{\text{old}}+{\lambda }_{j}\left(\frac{{w}_{ij}+{w}_{\text{elite},j}}{2}\right)-{W}_{hj}^{\text{old}},

where *W*_*i*_ defines the target solution in the current iteration, the term *i* is achieved based on the roulette wheel mechanism, i.e., the better solution will have more chance to be selected as *W*_*i*_, *W*_*hj*_ describes the *j*th member of *W*_*h*_, *W*_elite_ describes the position of the elite stored in the cycles, {\lambda }_{j} represents the attraction power in the target bower (solution) that is achieved by the following equation:

(8) {\lambda }_{j}=\frac{\alpha }{1+{o}_{i}},

where the term \alpha stands for the greatest size of step using the target solution and *o*_*i*_ describes the probability obtained by {\text{Prob}}_{i}.

#### 2.2.4 Mutation

While building the bower, the male satin bowerbird might be attacked or even totally ignored by other animals. During the bower construction, stronger males destroy the weaker males’ bower or steal their materials. Therefore, arbitrary changes have been adopted at the end of each cycle with a specific probability. This modification is implemented to *W*_*hj*_ with a specific probability. A normal distribution (*L*) has been adopted with variance {\alpha }^{2}and average of {W}_{hj}^{\text{old}} as follows:

(9) {w}_{hj}^{\text{new}}\sim L({w}_{hj}^{\text{old}},{\alpha }^{2}),

(10) L({w}_{hj}^{\text{old}},{\alpha }^{2})={w}_{hj}^{\text{old}}+(\alpha \times L(0,1)).

The proportion of space width is represented as a value of *α* that is evaluated in equation (16).

(11) \alpha =y\times ({\text{Var}}_{\max }-{\text{Var}}_{\min }),

where *y* describes the variance ratio among lower and upper ranges, and var_max_ and var_min_ represent the upper and the lower limits assigned to the variable.

The population that is achieved after modifications at the end of every cycle and the old population is evaluated. The populations are then combined and sorted after evaluating, and the new population has been generated. Once the termination condition is satisfied, the algorithm is stopped.

### 2.3 Median filtering

Medical images are often damaged by noise when acquired and passed on. The purpose of image noise reduction methods is to eliminate such noise while maintaining the essential features of the image as much as possible. Dermoscopy imaging is a widely used medical imaging process, as it is economically, relatively safe, and adaptable. However, one of its major disadvantages is the poor quality of images due to spot noise. Spot noise is a disgrace, as it undermines image quality and affects single interpretation and recognition operations. Consequently, point refining is a key stage for the function’s extraction, analyzing and recognizing medical images. There are several useful methods for noise removal from the medical images. Median filter due to its specificity has the most application in medical image noise removal [31,32]. The main idea behind median filtering is to present an *m* × *n* neighborhood to assemble all neighborhoods in the ascending order, select the median value of the ordered numbers, and replace the central pixel. This strategy is modeled by the following equation:

(12) {y}_{(m,n)}=\text{median{}{x}_{(i,j)},(i,j)\in C\text{},}

where *C* represents the centered neighborhood around location (*m*, *n*) of the image.

In this research, a median filter is adopted for digital noise removal in the skin cancer dermoscopy image, in which a filter mask with a size of 3 × 3 is employed.

## 3 Medical skin cancer diagnosis based on optimized CNN

The main contribution of this study is to adopt the SBO algorithm for optimal justification of the number of hyperparameters in CNN for the skin cancer diagnosis application. To avoid system error, the minimum range is considered to be 2 and the maximum range is considered equal to the size of sliding window. The reason for selecting 2 as the minimum range is that the allowed minimum value for the maximum pooling is such that there is no lower size. It is important to note that the inequality constraint of this problem is that the value of the sliding window should be smaller than the input data. In this study, the population for bowerbirds is considered 200. Table 1 indicates the architecture of the suggested CNN.

**Table 1:** Architecture of the suggested CNN

Layer #	Layer name	Properties
1	Input layer	Input image patch size: 256 × 256 × 3
2	Convolutional	Blocks of the size: 11 × 11
3	ReLU	—
4	Max pooling	Pool size 2 × 2
5	Convolutional	Blocks of the size: 7 × 7
6	ReLU	—
7	Max pooling	Pool size 2 × 2
8	Convolutional	Blocks of the size: 3 × 3
9	ReLU	—
10	Dropout	Dropout ratio: 0.6
11	Fully connected	1 × 256
12	Softmax	—

**Figure 3:**
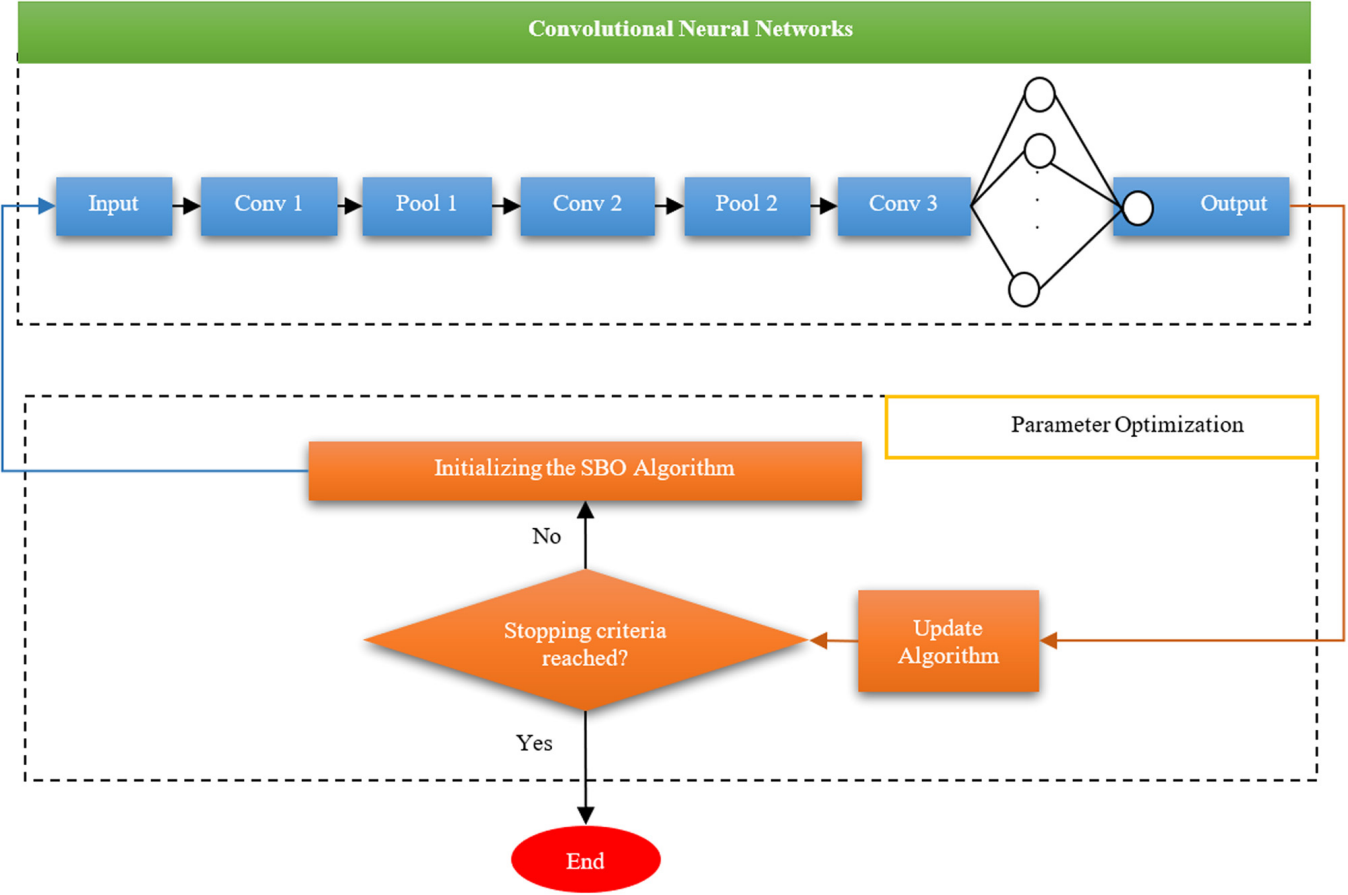
The optimized structure of the SBO-based CNN.

As mentioned earlier, the cost function is the half-value precision of CNN. After initializing, the position of each bowerbird has been updated based on the SBO algorithm. Once the stopping criteria is reached, the optimization process will be stopped. In this study, the weights and the biases of the CNN are chosen for optimization, i.e.,

(13) W=\{{w}_{1},{w}_{2},\ldots ,{w}_{p}\},

(14) A=\{{a}_{1},{a}_{2},\ldots ,{a}_{A}\},

(15) {w}_{n}=\{{w}_{1n},{w}_{2n},\ldots ,{w}_{Ln}\},

(16) \begin{array}{l}{b}_{n}=\{{b}_{1n},{b}_{2n},\ldots ,{b}_{Ln}\}\\ l=1,2,\ldots ,L\\ n=1,2,\ldots ,N,\end{array}

where *N* describes the overall number of optimization individuals, *L* represents the overall number of layers, *l* determines the layer index, *n* represents the number of bowerbirds, and *w*_in_ represents the value of the weight in the *i*th layer. The optimized configuration of the SBO-based CNN is shown in Figure 3.

The error between the desired output and the network output is calculated by the following equation:

(17) E=\frac{1}{Ns}{\mathop{\sum }\limits_{i=1}^{Ns}\mathop{\sum }\limits_{j=1}^{k}({d}_{ji}-{o}_{ji})}^{2},

where *k* and *Ns* represent the output layers quantity and training samples, respectively, and *o*_*ji*_ and *d*_*ji*_ describe the CNN output and the desired output.

**Figure 4:**
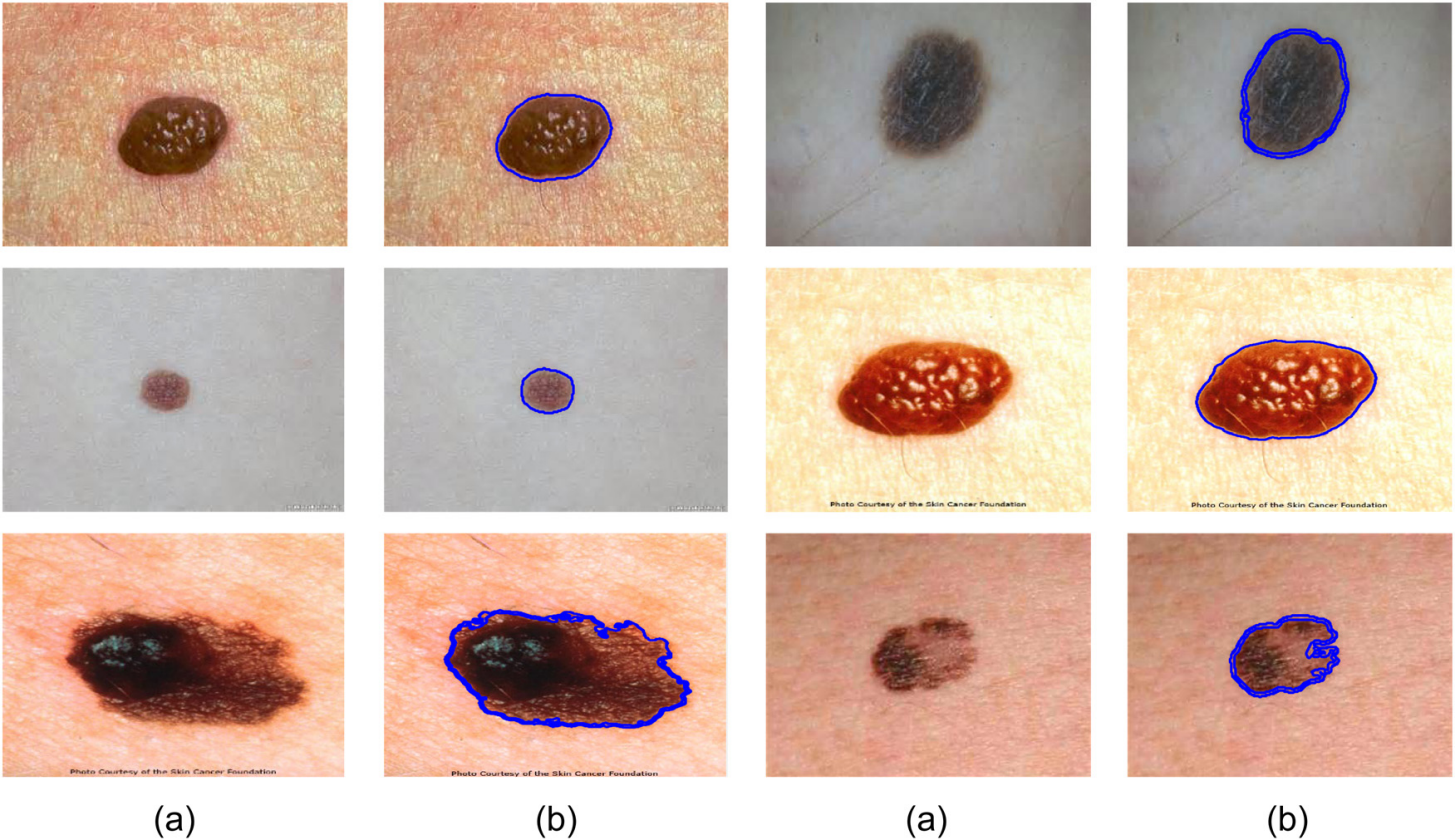
The results of cancer diagnosis for some examples by the SBO-based CNN. (a) before process, (b) after process.

Since the gradient descent has been trapped into the local minimum in some cases, the SBO algorithm has been adopted [33,34,35]. Figure 4 shows the results of cancer diagnosis for some examples by the SBO-based CNN.

The confusion matrix for the system is 2\times 2 as the American Cancer Society (ACS) database is selected and divided into two classes: the first 40% plus the second 60% with noise (ACS1) and the second 60% plus the first 40% with noise (ACS2). This matrix has been achieved by adding two confusion matrices on the aforementioned two categories of the dataset. We note a good percentage of correctly classified ACS1 and ACS2. The number of correctly classified ACS2 at a first glance looks quite lower than ACS1, which is due to the small number of high-quality images in ACS2 compared with that of ACS1 (Table 2).

**Table 2:** Confusion matrix for the system

	ACS1	ACS2
ACS1	570	9
ACS2	7	320

## 4 Feature extraction and selection based on SBO

The process of extracting more precise information from the cancer area is called feature extraction, i.e., the purpose of feature extraction in this step is to use this trend as a method for skin cancer detection after segmenting the skin cancer area. For more clarification, the original image is a raw datum with enormous information. This reason increases the complexity of image processing and is time-consuming. A good method here is to use feature extraction for simplifying the process. Several features have been introduced for the extraction of image features. In this study, statistical features, geometric features, and texture features are adopted, which are listed in Table 3.

**Table 3:** Adopted features in this study

Parameter	Equation	Parameter	Equation
Perimeter	\mathop{\sum }\limits_{i=1}^{M}\mathop{\sum }\limits_{j=1}^{N}{B}_{p}(i,j)	Correlation	\mathop{\sum }\limits_{i=1}^{M}\mathop{\sum }\limits_{j=1}^{N}\frac{p(i,j)-{\mu }_{r}{\mu }_{c}}{{\sigma }_{r}{\sigma }_{c}}
Area	\mathop{\sum }\limits_{i=1}^{M}\mathop{\sum }\limits_{j=1}^{N}p(i,j)	Mean	\frac{1}{MN}\mathop{\sum }\limits_{i=1}^{M}\mathop{\sum }\limits_{j=1}^{N}p(i,j)
Solidity	\text{Area/Convex}\hspace{.5em}\text{area}	Entropy	-\mathop{\sum }\limits_{i=1}^{M}\mathop{\sum }\limits_{j=1}^{N}p(i,j)\log \hspace{.25em}p(i,j)
Elongation	\frac{2\sqrt{\text{Area}}}{a\sqrt{\text{π}}}	Variance	\frac{1}{MN}\mathop{\sum }\limits_{i=1}^{M}\mathop{\sum }\limits_{j=1}^{N}(p(i,j)-\mu )
Rectangularity	\frac{\text{Area}}{b\times a}	Standard deviation	{\text{variance}}^{\frac{1}{2}}
Irregularity index	4\text{π}\times {\text{Area/Perimeter}}^{2}	Invariant moments	{\varphi }_{1}={\eta }_{20}+{\eta }_{02}
			{\varphi }_{2}={({\eta }_{20}-{\eta }_{02})}^{2}+4{\eta }_{11}^{2}
			{\varphi }_{3}={({\eta }_{30}-3{\eta }_{12})}^{2}+{(3{\eta }_{21}-{\mu }_{03})}^{2}
Form factor	\frac{\text{Area}}{{a}^{2}}		
*E*_centricity_	2{a}^{-1}{({a}^{2}-{b}^{2})}^{0.5}	Energy	\mathop{\sum }\limits_{i=1}^{M}\mathop{\sum }\limits_{j=1}^{N}{p}^{2}(i,j)
Contrast	\mathop{\sum }\limits_{i=1}^{M}\mathop{\sum }\limits_{j=1}^{N}{p}^{2}(i,j)	Homogeneity	\mathop{\sum }\limits_{i=1}^{M}\mathop{\sum }\limits_{j=1}^{N}\frac{p(i,j)}{1+|i-j|}

{B}_{p} describes the length of external side for the boundary pixel, *MN* represents the image size, p(i,j) defines the value of the pixels intensity at location (*i*, *j*), *a* is the major axis and *b* is the minor axis, \mu describes the mean value, and \sigma stands for the standard deviation.

Nevertheless, some of the explained features have more effect and some others have less effect with low information from the image. In this study, an optimized procedure is used for better selection of the useful features from the image. The optimal feature selection here is performed by SBO. The fitness function of the feature selection is considered as follows:

(18) \text{Fitness}=\frac{(\text{TP}\times \text{TN})-(\text{FP}\times \text{FN})}{\sqrt{((\text{TN}+\text{FP})\times (\text{TP}+\text{FP})\times (\text{TP}+\text{FN})\times (\text{TN}+\text{FN}))}},

where the main parts of the function are false positive (FP), false negative (FN), true positive (TP), and true negative (TN). The main idea based on the proposed method is to minimize the fitness value in equation (18) by optimal selection of the features. After optimal feature extraction of the medical images, a classification method is required to determine the cancerous and the healthy cases. To understand how the features can help easier classification, some significant example features for both cancerous and healthy images are given in Table 4.

**Table 4:** Some significant example features for both cancerous and healthy images

Parameter	Cancerous				Healthy			
	#1	#2	#3	#4	#1	#2	#3	#4
Correlation	0.9915	0.9886	0.9872	0.9505	0.9905	0.9898	0.9864	0.9779
Area	0.3847	0.4492	0.5133	0.9389	0.3988	0.4266	0.4996	0.6086
Energy	0.6419	0.6348	0.5746	0.6185	0.6538	0.6320	0.6193	0.6510
Form factor	0.1205	0.1811	0.2253	0.6428	0.1096	0.1328	0.1973	0.1308
Eccentricity	0.1215	0.1886	0.1872	0.1505	0.1205	0.1798	0.1864	0.2779
Homogeneity	0.9974	0.9964	0.9953	0.9843	0.9972	0.9968	0.9955	0.9934
Contrast	0.1480	0.2018	0.2635	0.8817	0.1591	0.1820	0.2496	0.3704

## 5 Image classification

In this study, SVM has been employed for feature classification. The method includes a set of points in the *n*-dimensional space, which indicates and sorts the class boundaries and can be changed by replacing with one of two cases. SVM selects the optimal decision surface by the following formula:

(19) y=\mathrm{sgn}\left(\mathop{\sum }\limits_{i=1}^{N}{y}_{i}{\alpha }_{i}K(x,{x}_{i})+b\right),

where {x}_{i} represents the training set vector number *i*, *x* describes a d-dimensional test set vector, *N* defines the training set numbers, K(x,{x}_{i}) describes a kernel function, *y* describes a class label in the range [−1, 1], and \alpha =\{{\alpha }_{1}\ldots {\alpha }_{N}\}, and *b* determines the model parameters. Due to the high potential of SVM, it is adopted for classifying the skin cancer dermoscopy image into two categories of cancerous and healthy groups.

## 6 The database

**Figure 5:**
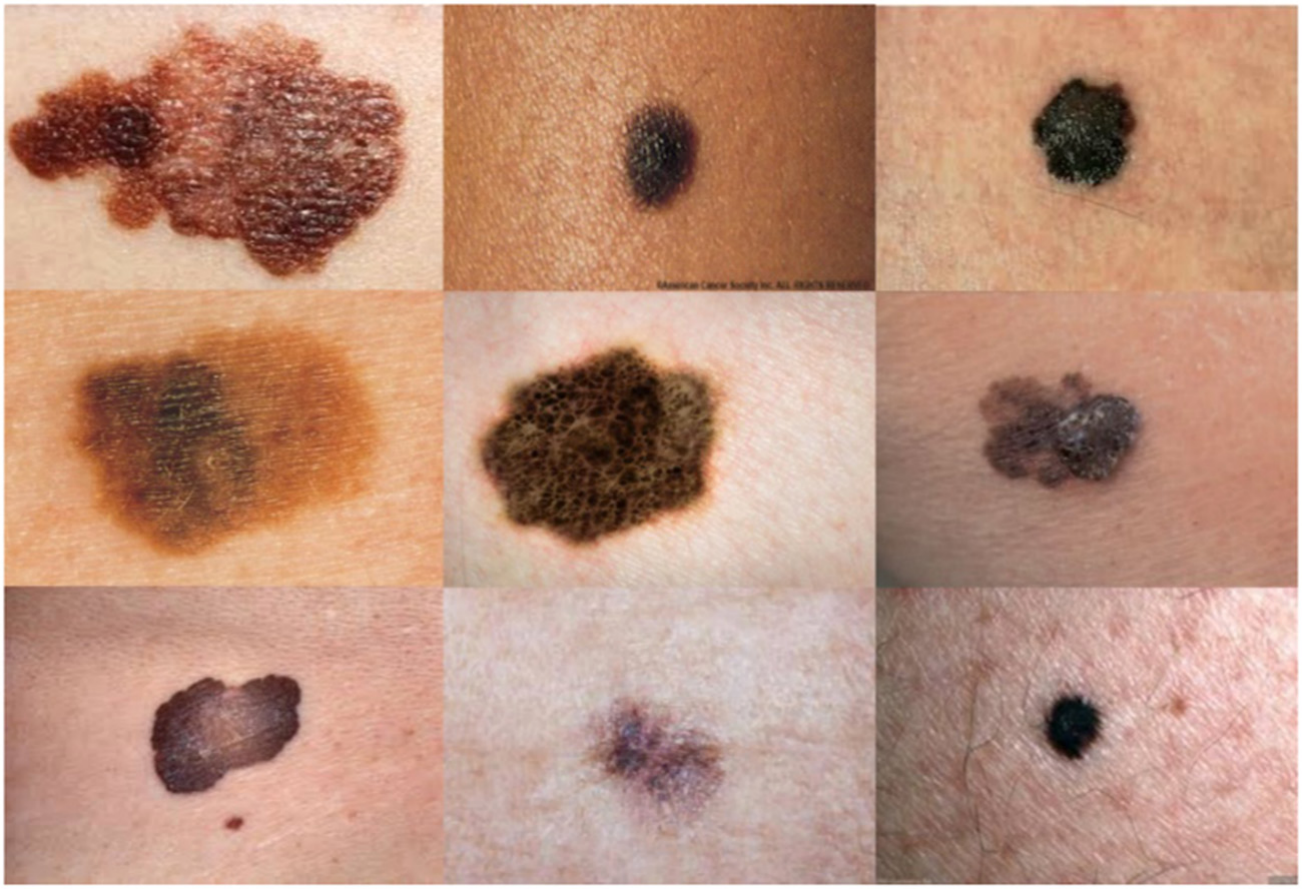
Some examples of the used dataset from ACS.

There are several datasets for skin cancer detection and the validation of different methods. This research adopts the ACS dataset. This dataset contains 68 pairs of XLM and TLM images that are obtained by the same Nevoscope device. All the images have been resized into 256 × 256 pixels to reduce the system computational complexity [36]. Figure 5 shows some examples of the used dataset from ACS.

## 7 Simulation results

This section validates and discusses about the results of the proposed skin cancer diagnosis system. The method has been simulated based on MATLAB software version R2018a with a 32 GB Ram, Core i7Intel processor and two SLI GeForce Titan GPUs. The method is performed for the ACS skin cancer dataset for performance analysis of the system. The procedure of the method is given as follows:

1. Apply median filtering for noise elimination of the input dermoscopy image.
2. Apply optimized CNN based on the SBO algorithm for cancer area segmentation.
3. Extract important features from the segmented image.
4. Apply feature selection based on the SBO algorithm.
5. Classify cancerous and healthy cases based on the SVM classification methodology.

For the proposed SVM classifier, 75% of data is considered for training and 25% is used for validation and testifying the method. Training stage for the network is considered by 8,000 iterations. The training step is repeated 15 times for achieving a robust analysis. The analysis is applied based on five performance indexes, including accuracy, specificity, positive predictive value (PPV), negative predictive value (NPV), and sensitivity, which are formulated as follows:

(20) \text{Accuracy}=\frac{\text{correctly}\hspace{.5em}\text{detected}\hspace{.5em}\text{cases}}{\text{total}\hspace{.5em}\text{cases}},

(21) \text{Specificity}=\frac{\text{correctly}\hspace{.5em}\text{detected}\hspace{.5em}\text{healthy}\hspace{.5em}\text{skin}\hspace{.5em}\text{cases}}{\text{total}\hspace{.5em}\text{healthy}\hspace{.5em}\text{skin}\hspace{.5em}\text{cases}},

(22) \text{PPV}=\frac{\text{correctly}\hspace{.5em}\text{detected}\hspace{.5em}\text{skin}\hspace{.5em}\text{cancer}\hspace{.5em}\text{cases}}{\text{detected}\hspace{.5em}\text{skin}\hspace{.5em}\text{cancer}\hspace{.5em}\text{cases}},

(23) \text{NPV}=\frac{\text{correctly}\hspace{.5em}\text{detected}\hspace{.5em}\text{healthy}\hspace{.5em}\text{skin}\hspace{.5em}\text{cases}}{\text{detected}\hspace{.5em}\text{healthy}\hspace{.5em}\text{skin}\hspace{.5em}\text{cases}},

(24) \text{Sensitivity}=\frac{\text{correctly}\hspace{.5em}\text{detected}\hspace{.5em}\text{skin}\hspace{.5em}\text{cancer}\hspace{.5em}\text{cases}}{\text{Total}\hspace{.5em}\text{skin}\hspace{.5em}\text{cancer}\hspace{.5em}\text{cases}}.

The final results are compared with ten different methods from the literature in terms of efficiency that contain a method [37] using semi-supervised system, some methods based on CNN, ResNet [38], a commercial tool [39], VGG-16[40], AlexNet [41], Inception-v3 [42], and LIN [43]. Table 5 indicates the efficiency results of the proposed method compared with the other state of art techniques.

**Table 5:** Efficiency analysis of the proposed method compared with the other state-of-art techniques

Method	Performance metric				
	Accuracy	Sensitivity	Specificity	NPV	PPV
LIN [43]	0.89	0.92	0.90	0.93	0.82
AlexNet [41]	0.83	0.84	0.62	0.85	0.67
VGG-16[40]	0.87	0.91	0.87	0.91	0.79
Spotmole [39]	0.69	0.84	0.60	0.86	0.59
Ordinary CNN	0.82	0.82	0.80	0.87	0.76
ResNet-50 [38]	0.83	0.87	0.80	0.84	0.72
ResNet-101 [38]	0.86	0.85	0.78	0.90	0.76
Inception-v3 [42]	0.85	0.86	0.66	0.73	0.64
MED-NODE texture descriptor [37]	0.79	0.64	0.87	0.80	0.77
MED-NODE color descriptor [37]	0.75	0.77	0.74	0.84	0.66
Proposed method	0.95	0.95	0.92	0.96	0.87

**Figure 6:**
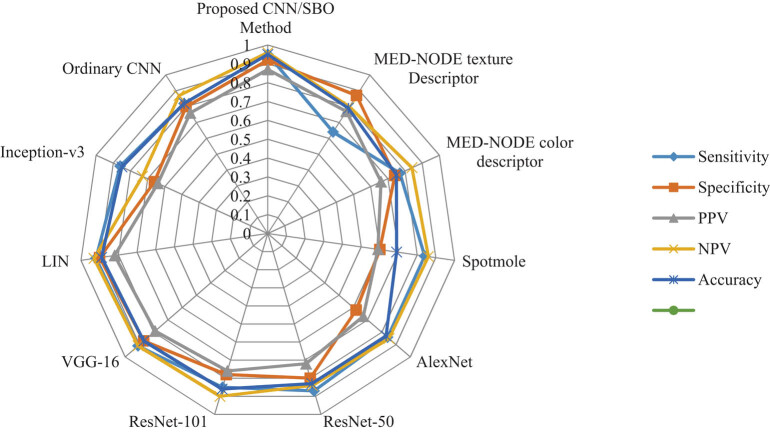
The Radar plot of the classification rate of skin cancer classification based on the presented technique and the other methods from the literature and its efficiency is indicated by the confusion matrix.

Figure 6 shows the radar plot of the classification rate. As shown in Table 2 and Figure 6, the presented procedure has the highest accuracy compared with the other methods from the literature.

## 8 Conclusions

In the present study, an automatic computer-aided methodology was proposed for the skin cancer detection from dermoscopy images. To achieve higher accuracy for the diagnosis, the input images were first denoized based on a median filter. Afterward, a new optimized method was presented for cancer area segmentation using a developed CNN based on the SBO algorithm to classify the cancerous region from the background. Then, numerous features were extracted from the segmented images. To simplify the classification process, an optimal method based on the SBO algorithm was adopted for optimal feature selection and decreasing the order of features. To do the final classification, the extracted features from the images were classified based on an optimized SVM classifier using the SBO algorithm. The design was then applied to the ACS database and was compared with ten state-of-art methods to indicate the system efficiency. Final results showed that by analyzing accuracy, specificity, NPV, sensitivity, and PPV, the presented technique has better performance than the compared methods. The future work will be about using an improved model of the proposed method to achieve good results for breast cancer detection.
